# A Rare Case of Giant-Cell Tumor of Hand in a Young Male

**DOI:** 10.7759/cureus.21408

**Published:** 2022-01-19

**Authors:** Siddharth Yadav, Shivani Singhal, Shivam Patel, Shubham Jaiswal, Roshni Mishra

**Affiliations:** 1 Orthopaedics, Dr. D.Y. Patil Medical College, Pune, IND; 2 Pathology, Pandit Bhagwat Dayal Sharma Post Graduate Institute of Medical Sciences, Rohtak, IND; 3 General Surgery, Dr. D.Y. Patil Medical College, Pune, IND; 4 Dermatology, Dr. D.Y. Patil Medical College, Pune, IND

**Keywords:** giant-cell tumor of bone, orthopedic oncology surgery, metacarpal tumor, rare tumor, rare bone tumor, histopathology examination

## Abstract

Giant-cell tumor (GCT) of the bone affecting the hand is a rare lesion that is usually diagnosed at an advanced stage and has a high rate of recurrence. In the current literature, GCT is described as a predominantly osteoclastogenic stromal cell tumor of mesenchymal origin. It is composed of three cell types: the neoplastic GCT stromal cells; mononuclear monocyte cells; and multinucleated giant cells. Clinical imaging is basic for the diagnosis of a GCT. This tumor within the hand tends to be less eccentric and most often central. GCT of metacarpals is noted to be a rare location, with the incidence being as low as 2%. GCT on hand as compared to other sites is locally more aggressive, grows faster, and has a higher recurrence rate.

A 22-year-old male patient presented with swelling over the left hand for 7 months, spontaneous in onset, gradually progressive in size, and painfully restricting the joint movement, with no history of fall or trauma. On examination, diffuse swelling of size 5 × 5 × 3 cm was tender on palpation, restricting the movement at the 4th metacarpophalangeal joint. A plain radiograph followed by an MRI scan revealed a Campanacci’s Grade III GCT of the 4th metacarpal. An open biopsy showed an expanded and lytic mass with areas of hemorrhage and necrosis. There were few mitotic figures and the tumor was diagnosed to be a GCT. On surgical resection, friable tumor tissue was noted over the region of the entire 4th metacarpal except for the base. The patient was managed by surgical intralesional excision of the mass, followed by Kirschner-wire fixation and reconstruction with synthetic bone graft. The excised tissue was sent for histopathological examination. The patient was followed up at regular intervals, with initial splinting, followed by wire removal at 6-week post-op, and with adequate physiotherapy, as tolerated by the patient. On a 3-month follow-up, the range of motion had returned to a functional level, with good uptake of graft, and no other complications.

GCT of the hand is a rare presentation of the disease and requires meticulous workup, including a thorough clinical exam, hematological, radiological, and pathological workup. The various treatment modalities described in the literature for GCTs are curettage alone, curettage and bone graft, en-bloc resection, amputation, and resection with reconstruction, but curettage alone or curettage with bone graft is not effective even for GCTs of long bones and hand, too. Such a procedure creates a skeletal void and hence furthers the need for a challenging reconstructive procedure requiring reconstruction using autograft, allograft, or silastic (synthetic) implant.

## Introduction

Giant-cell tumor (GCT) of the hand is an uncommon lesion that is mostly identified late in its progression and has a high recurrence rate. Giant-cell tumor of bone (GCTB) is now classified as a primarily osteoclastogenic stromal cell tumor of mesenchymal origin in the literature. The neoplastic mononuclear stromal cells, mononuclear monocyte cells, and multinucleated giant cells are the three cell types that make up this tumor. The use of clinical imaging for the diagnosis of GCTB is essential. This type of tumor in the hand is usually less eccentric and more central.

Averill et al. divided GCTB into three grades: grade-1, quiescent, is a static form with limited cortex involvement; grade-2, active, is characterized by a thinned and bulged cortex, and grade-3, aggressive, is characterized by a lesion that penetrates the cortex and includes a soft tissue component [1].

In the literature, various therapeutic approaches for GCTB in the hand have been described: curettage; curettage with or without adjutants, followed by bone graft or methylmethacrylate bone cement packing; extensive excision and repair; amputation and disarticulation.

## Case presentation

A 22-year-old male was complaining of pain and swelling in his left hand and limited range of motion in his ring finger for 7 months. There was no mention of a fall or any trauma. On inspection, there was a globular swelling measuring 5 × 5 × 3 cm across the dorsum of the left hand, as well as pain. The pain was dull and agonizing, and it was eased in part by immobilization and the use of painkillers. There were no signs of a neurovascular problem. The region’s sensations were unaffected. There were no obvious deformities found. On palpation, there was a local temperature rise.

Investigations

**Figure 1:**
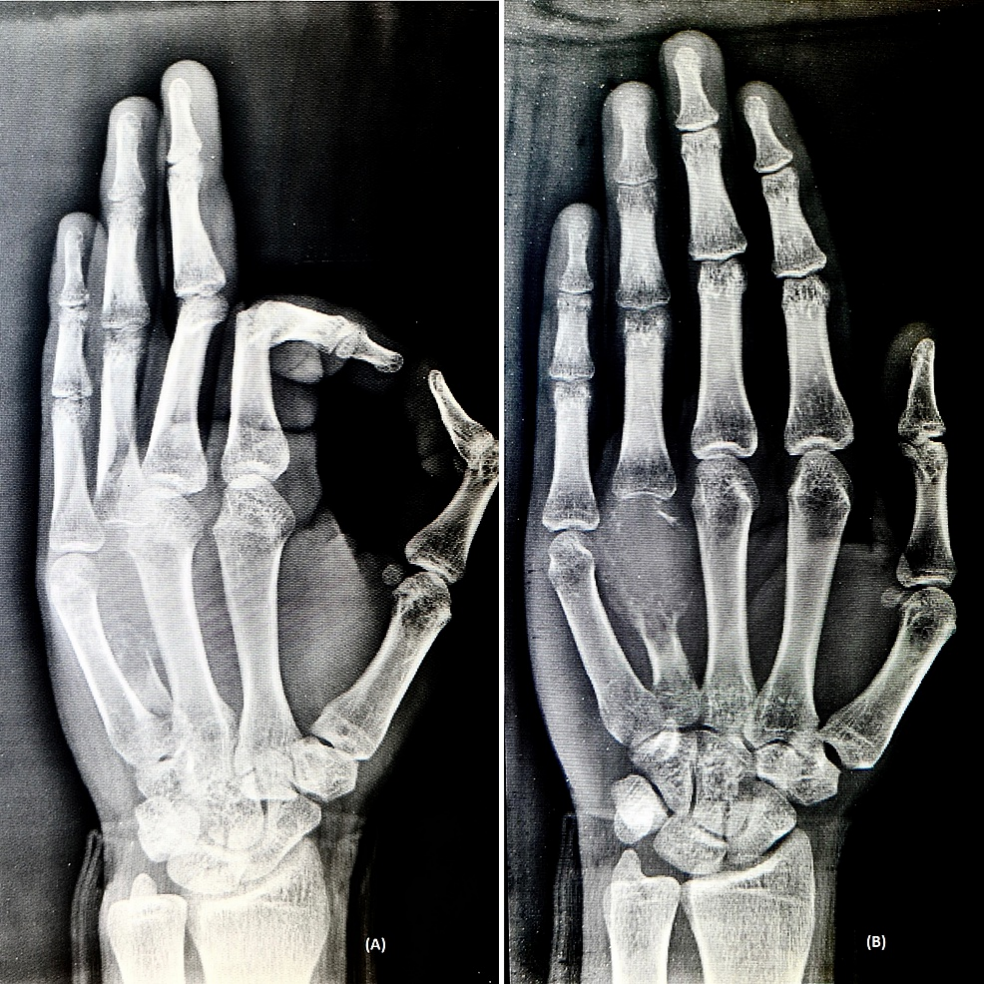
Radiograph of the left hand in A) oblique view and B) anteroposterior view.

A plain radiograph was ordered, which showed a lytic and expansile lesion of the shaft of the 4th metacarpal of the left hand (Figure 1).

**Figure 2:**
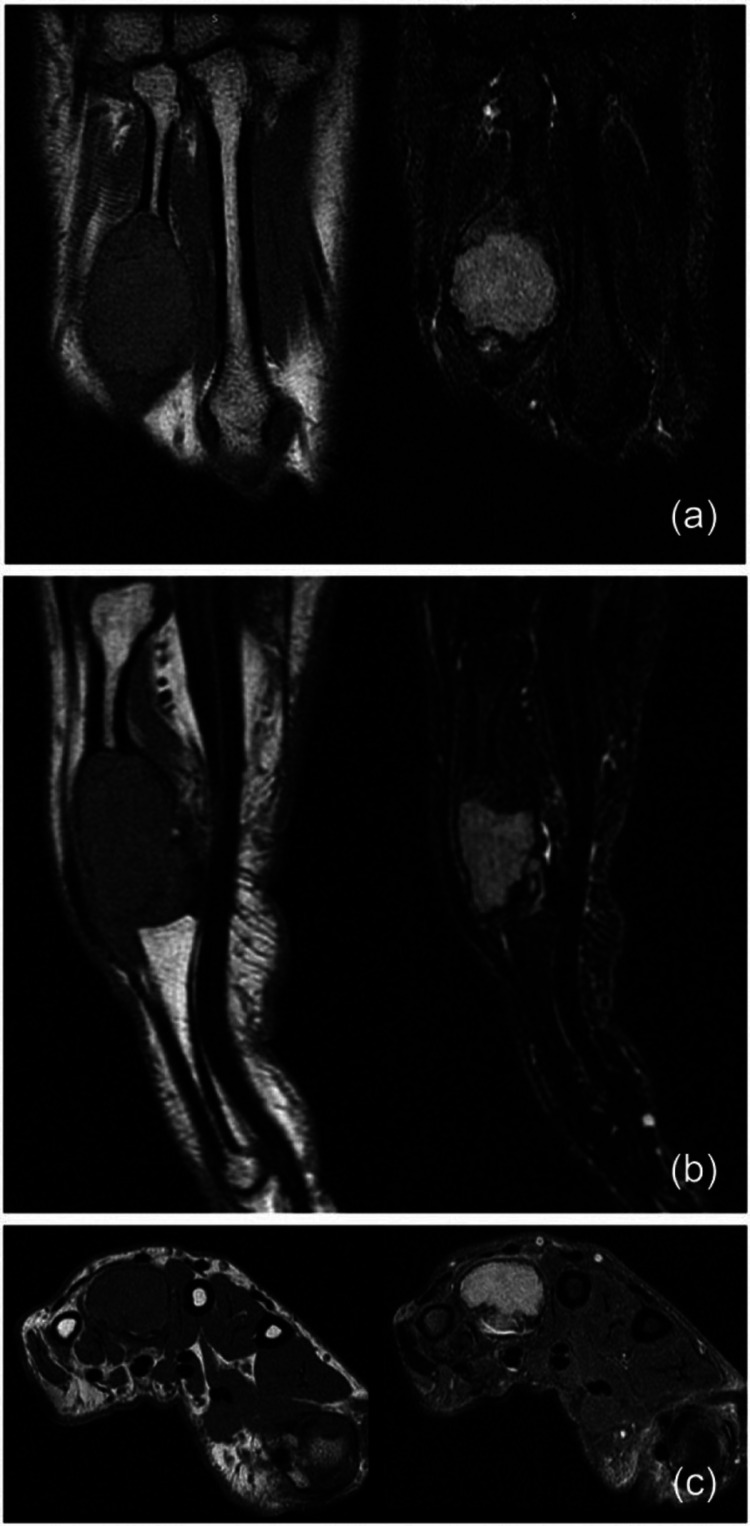
MRI cuts showing multiple lytic lesions along with a pathological fracture of the left 4th metacarpal.

For further evaluation, an MRI of the left hand was done, which showed an expansile, lytic, intramedullary lesion in the shaft of the 4th metacarpal with pathological fracture of both cortices, findings which were suggestive of Campanacci’s grade III GCT of the 4th metacarpal bone (Figure 2).

Serum calcium, phosphorus, and alkaline phosphatase levels were all within normal limits. An open biopsy revealed an enlarging, lytic tumor with hemorrhage and necrosis. The tumor was identified as a GCT low-grade since there were few mitotic features.

Histology findings

**Figure 3:**
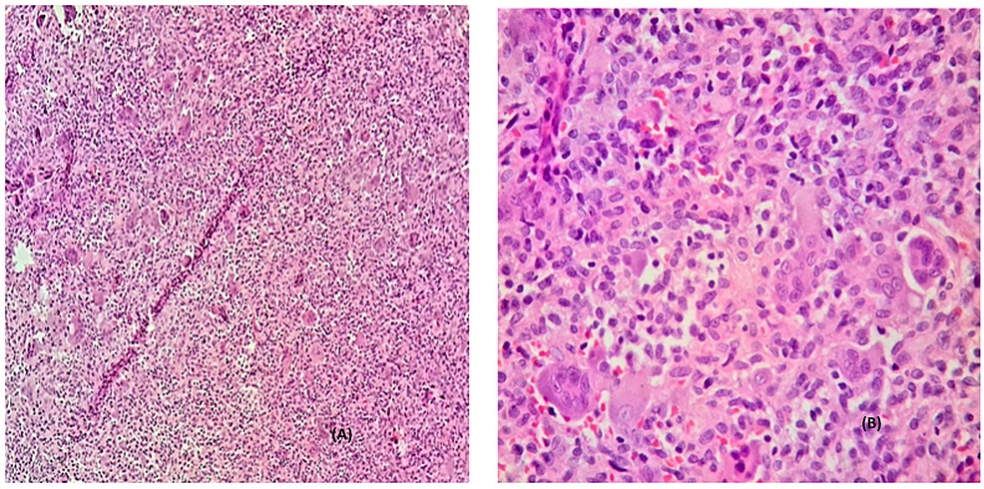
Histological findings conformed to the diagnosis of GCT. A) Highly cellular lesion with multinucleated giant cells and mononuclear stromal cell component (H&E, 200×). B) High-power view shows multinucleated giant cells and mononuclear oval to spindle stromal cells with mitotic figures (H&E, 400×). GCT: giant-cell tumor; H&E: hematoxylin and eosin

Histological findings conformed to the diagnosis of GCT revealing multinucleated giant cells and mononuclear stromal cells. These stromal cells were round to oval to spindle in shape with eosinophilic cytoplasm and nuclei with dispersed chromatin (Figure 3).

**Figure 4:**
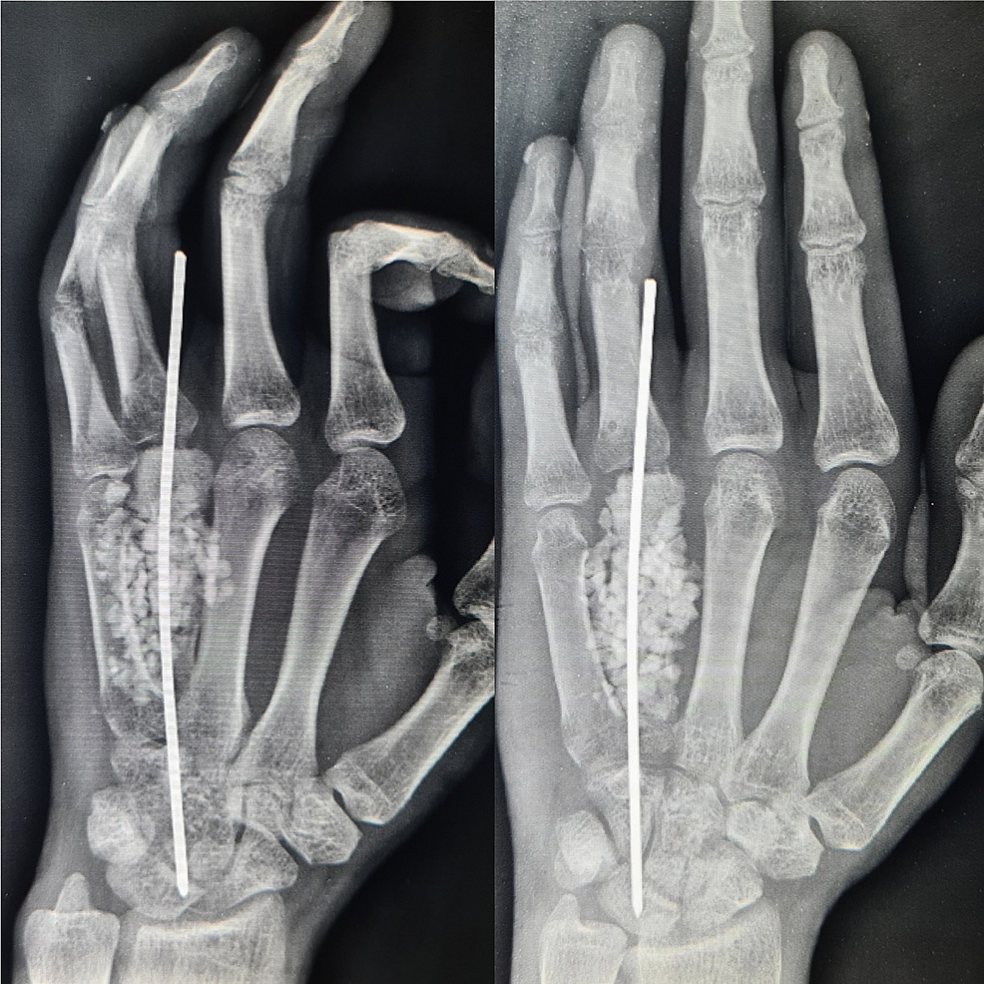
Post-op radiograph showing the intramedullary Kirschner-wire following tumor excision and augmentation of defect with bone graft.

After a thorough preoperative examination and assessment of operative fitness, the patient was scheduled for excision of the tumor mass, intramedullary fixation with a Kirschner-wire, and augmentation with a synthetic bone graft (Figure 4), the operation was carried out without any complications.

## Discussion

GCT of the hand bone is a rare but distinct and more aggressive lesion than GCT of the rest of the skeleton, with a higher frequency in Asian populations than in western populations [1]. GCT of the metacarpals is a rare occurrence, having an incidence of less than 2% [2]. In comparison to other sites, GCT of the hand is more aggressive locally, develops faster, and has a higher recurrence rate [3]. The early radiological appearance of GCT can be mistaken for enchondroma, however, the more aggressive nature of hand GCT can help distinguish the two entities.

Primary GCT of hand bones varies from GCT of other long bones in that it is more usually seen in the diaphyseal region as opposed to the epiphyseal region of long bones [4]. Pain, swelling, and soreness are common presenting complaints, along with a reduction of range of motion if the adjacent joint is also affected. However, some patients have come with a pathological fracture but no prior medical history [5]. When GCTB is suspected, a plain radiograph is the first and most straightforward investigation. It typically displays an expanding zone of radiolucency as well as a lesion that is either well or weakly marginated without a sclerotic boundary. Using radiological evidence, Campanacci et al. described three levels of illness. A favorable link between aggressiveness and a high recurrence rate has been documented at the higher grade level. A well-defined margin with a thin rim is described as Grade I. Grade II lesions affect a greater region and extend to the cortical layer without the shattering that characterizes grade III [5,6].

The imaging modality of choice for diagnosing GCT is MRI, which aids in determining the size of the lesion as well as intra- and extra-osseous dissemination. GCT imaging findings can resemble a variety of different bone disorders, particularly enchondroma, which is more frequent in the hand [7]. As a result, tissue diagnosis is required for confirmation. In general, GCTB is dark brown in color and has a soft to firm firmness. Fibrosis and osteoid development can be noticed in this area. There are blood-filled cystic lesions visible. The typical multinucleated giant cells with neoplastic mononuclear stromal cells are the histological hallmark of GCT of bone. Without nuclear atypia, mitotic activity can be observed [6,8]. Finding diagnostic regions for GCT is essential for histologically distinguishing between distinct giant cell-rich lesions. In some cases, due to a lack of material, a differential diagnosis with other giant cell-rich lesions is impossible [9]. As a result, while making a diagnosis, clinical presentation, imaging, and tissue examination results should all be considered [7]. As a preoperative prerequisite and to assess for pulmonary metastases, a chest radiograph should be frequently requested. CT scans can also aid in the detection of such lesions [6,10].

Curettage, curettage and bone graft, en-bloc resection, amputation, and resection with reconstruction are some of the treatment options mentioned in the literature [1], however, curettage alone or curettage with bone graft is ineffective even for giant cell tumors of the long bones and hand [1,6]. Such a surgery leaves a skeletal void, necessitating a difficult reconstructive treatment including autograft, allograft, or silastic (synthetic) implant restoration [7]. Chemical adjuvants, such as phenol, can be utiliszed as cytotoxic agents. In situations with uncontrolled recurrence, en-bloc resection is reserved as a last option [11]. Furthermore, numerous cavitary adjuvant therapy techniques are now frequently used to achieve acceptable local control, with a recurrence incidence of 6-25% [11,12].

The histologic diagnosis helps solve the problems, and the ﬁnding of diagnostic areas for GCT is mandatory. Nevertheless, occasionally, in the presence of insufﬁcient material, the differential diagnosis with other giant cell-rich lesions may be impossible. GCTs of the hand and foot appear to behave more aggressively compared with other giant cell-rich lesions and also compared with the general behavior of GCTs, although the cases in our series were few. The histologic diagnosis helps solve the problems, and the ﬁnding of diagnostic areas for GCT is mandatory.

GCT is well-known for its tendency to recur, with the highest frequency occurring in the first 24 months [1]. A recurrent case management algorithm, on the other hand, is comparable to that of the main GCT. On a 3-month follow-up, the range of motion had returned to a functional level, with good uptake of the graft, and no other complications were observed. The patient was further followed up at 6 monthly intervals. No recurrence was noted on subsequent follow-ups, with an acceptable and satisfactory range of motion achieved by the patient. A routine chest radiograph imaging was done to rule out any metastasis, and no signs of metastasis were appreciated on subsequent follow-ups.

## Conclusions

GCT of metacarpal bone presents as a challenge to the surgeon because of the rarity of its occurrence. Through this case report, we intend to lay down principles of management of such rare cases and broaden the discussion on existing literature of this rare presentation of bone malignancy in younger aged patients.

Throughout the treatment duration, an algorithm for surgical technique, rehabilitation, and follow-up regime was planned keeping in mind the patient’s age and requirement and to give maximum functionality.
